# Intravenous rAAV2/9 injection for murine cochlear gene delivery

**DOI:** 10.1038/s41598-017-09805-x

**Published:** 2017-08-29

**Authors:** Seiji B. Shibata, Hidekane Yoshimura, Paul T. Ranum, Alexander T. Goodwin, Richard J. H. Smith

**Affiliations:** 1 Department of Otolaryngology - Head and Neck Surgery, Carver College of Medicine, University of Iowa, Iowa City, IA 52242 USA; 2 Molecular Otolaryngology and Renal Research Laboratories, Carver College of Medicine, University of Iowa, Iowa City, IA 52242 USA; 3 Department of Otorhinolaryngology, Shinshu University School of Medicine, Matsumoto, Nagano 390-8621 Japan; 4 Interdisciplinary Graduate Program in Molecular & Cellular Biology, The University of Iowa Graduate College, University of Iowa, Iowa City, IA 52242 USA; 5 Iowa Institute of Human Genetics, Carver College of Medicine, University of Iowa, Iowa City, IA 52242 USA

## Abstract

Gene therapy for genetic deafness is a promising approach by which to prevent hearing loss or to restore hearing after loss has occurred. Although a variety of direct approaches to introduce viral particles into the inner ear have been described, presumed physiological barriers have heretofore precluded investigation of systemic gene delivery to the cochlea. In this study, we sought to characterize systemic delivery of a rAAV2/9 vector as a non-invasive means of cochlear transduction. In wild-type neonatal mice (postnatal day 0–1), we show that intravenous injection of rAAV2/9 carrying an eGFP-reporter gene results in binaural transduction of inner hair cells, spiral ganglion neurons and vestibular hair cells. Transduction efficiency increases in a dose-dependent manner. Inner hair cells are transduced in an apex-to-base gradient, with transduction reaching 96% in the apical turn. Hearing acuity in treated animals is unaltered at postnatal day 30. Transduction is influenced by viral serotype and age at injection, with less efficient cochlear transduction observed with systemic delivery of rAAV2/1 and in juvenile mice with rAAV2/9. Collectively, these data validate intravenous delivery of rAAV2/9 as a novel and atraumatic technique for inner ear transgene delivery in early postnatal mice.

## Introduction

Hearing loss is the most common sensory impairment in humans. It impacts 1 of every 1000 newborns and in 70% of these babies has an underlying genetic etiology^1^. Current clinical treatment options for hereditary hearing loss are limited to sound amplification and cochlear implantation^2^. Although these interventions are nearly always beneficial, when compared to biological hearing performance outcomes are modest. To preserve biological hearing, targeted or personalized habilitation options that focus on preserving or even restoring hearing function by inner ear transgene delivery have gained interest.

**Figure 1:**
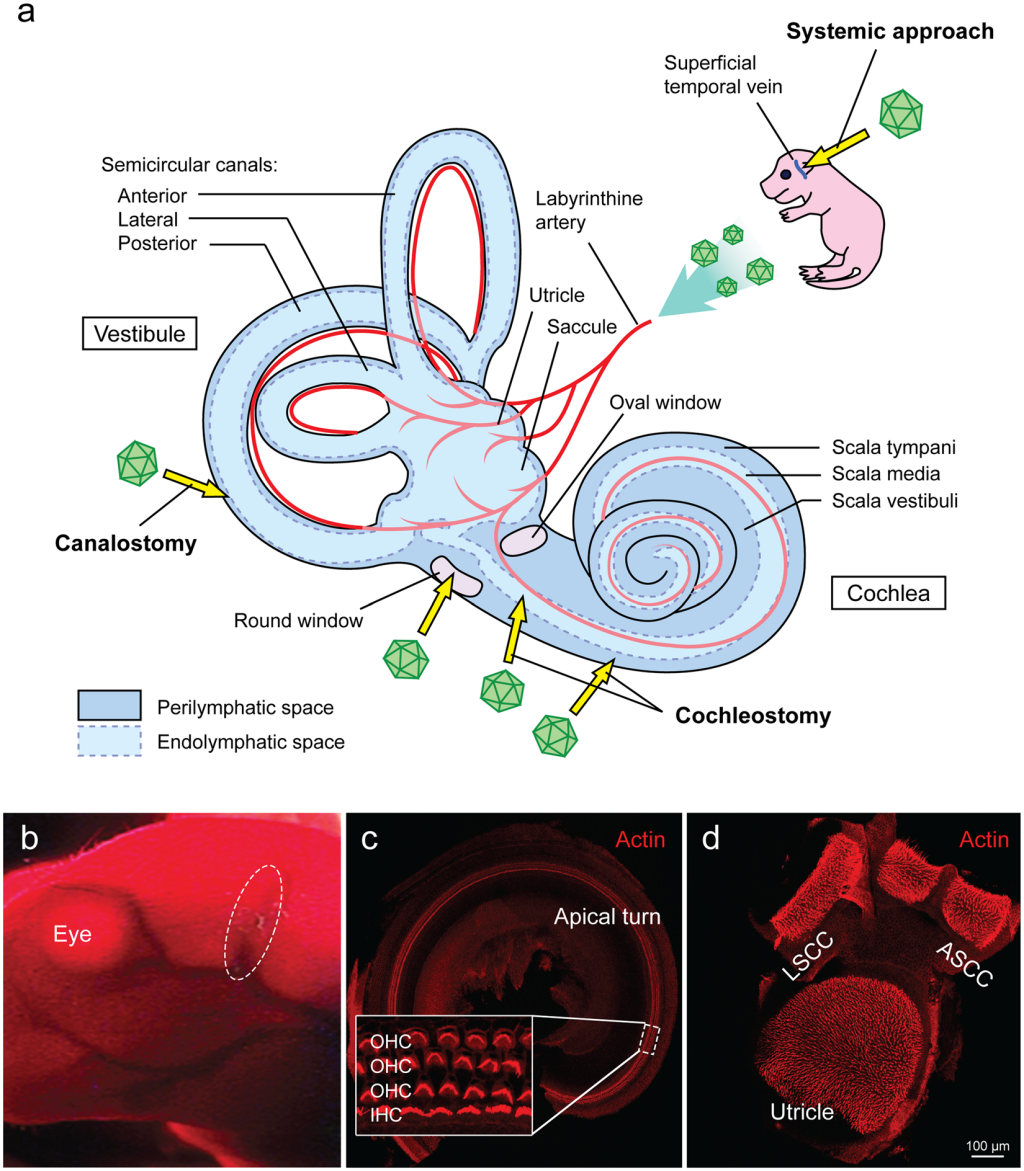
Inner ear schematic showing established delivery approaches and the systemic approach. Confocal images of representative whole-mount fluorescence-immunolabeling mouse cochlea and vestibule. (**a**) Vector delivery into the perilymph via a cochleostomy, canalostomy or trans-round window membrane; vector delivery into the endolymph via cochleostomy of the scala media space; systemic delivery via the superficial temporal vein. (**b**) Image showing a P0–1 pup after cryoanesthesia under infrared light. The superficial temporal vein for injection is shown in the dotted circle. (**c**) Representative confocal image of mouse cochlea showing three rows of outer hair cells (OHCs) and one row of inner hair cells (IHCs). (**d**) Representative confocal image of mouse vestibule showing utricle, anterior semicircular canal (ASCC) and lateral semicircular canal (LSCC). Note that red is phalloidin for labeling of filamentous actin.

One of the hurdles facing cochlear gene transfer is the delivery of a safe yet efficient amount of therapeutic to the cochlear epithelium. Because the mammalian inner ear is encased in the temporal bone, direct surgical intervention to access the membranous labyrinth is not trivial and can lead to unwanted side effects^3–5^. Established approaches to the perilymphatic or endolymphatic compartments include: (a) the perilymphatic approach, via a trans-round window membrane (RWM) injection^6–8^, cochleostomy to the scala tympani^9, 10^, (b) the endolymphatic approach, with a direct cochleostomy to the scala media^11, 12^, (c) or the semicircular canal canalostomy, which is likely a combination of the two pathways^13–15^ (Fig. 1a). While the perilymphatic approach is relatively safe and commonly used for cochlear implantation in humans^16^, the endolymphatic approach is complex and carries a high risk of inner ear damage making it clinically unfeasible, although efforts are on-going to establish an atraumatic approach to the endolymphatic space in neonatal and adult murine models^12, 17^. The development of a non-invasive and non-surgical method of transducing the inner ear may drive the translation of cochlear gene transfer into clinical practice.

To our knowledge, systemic gene transfer targeting the sensory and non-sensory epithelium in the inner ear has not been attempted (Fig. 1a). Reasons for this omission include potential systemic toxicities and two physiological barriers: the blood-brain barrier (BBB) and blood-labyrinth barrier (BLB), which obscure attempts to deliver larger molecules from the circulation into the target cells. The BBB is formed by endothelial tight junctions, pericytes, astrocytes and cellular basement membranes. Together, these structures comprise a barrier that precludes the entry of >98% of small molecules and most macromolecules^18^. The BLB has similar cellular structure and provides selective permeability by which to maintain inner ear homeostasis.

The recent finding that recombinant adeno-associated virus (rAAV) serotype 2/9 crosses the BBB after intravascular injection in postnatal and adult mice has impacted gene therapy studies targeting the CNS and retina^19–21^. For example, systemic gene therapy using rAAV2/9 is effective as a treatment for spinal muscular atrophy in P1–2 mice^22^. rAAV serotypes 2/1, 2/6, 2/7 and 2/10 also cross the BBB, however their CNS tissue transduction characteristics vary^23^. Other AAV serotypes, such as 2/2, 2/5 and 2/8, are BBB impermeable, although the precise mechanism for these differences is unknown^24^.

Direct surgical approaches have been used to characterize the transduction profiles of rAAV2/1 and 2/9 in neonatal/adult mouse and guinea pig cochleae however the ability of these vectors to traverse the BLB and transduce sensory and/or non-sensory cells in the inner ear epithelium has not been tested. Establishing the potential for systemic therapy is germane as there is increasing interest in delivering early postnatal preventative therapy to rescue hereditary deafness in mutant mice (specifically 0 to 48 hours after birth). In a recent proof-of-principle study, successful auditory restoration was achieved in mice with a targeted deletion of *VGLUT3* following neonatal trans-round window membrane injection of AAV1 carrying *VGLUT3* ^25^. Hair cell transduction was higher and auditory restoration lasted longer when treatment was initiated at an earlier time point (postnatal day 1–2, P1–2) as compared to a later time point (P10)^25^. Successes have also been reported with early interventions using antisense oligonucleotide and RNA interference to rescue or prevent hearing loss in mouse models of genetic deafness^26, 27^. Enhanced delivery of therapeutics to target cells of the neonatal ear is expected to result in even better performance outcomes.

In this study, we evaluated the transduction profile and efficiency of systemically introduced rAAV2/9 vectors expressing eGFP as a reporter gene in wild-type neonatal murine ears. Our results indicate that rAAV2/9 transduces auditory sensory epithelium in a binaural dose-dependent fashion without affecting auditory thresholds in clicks and pure tone frequencies measured at 8 kHz, 16 kHz and 32 kHz. Inner ear transduction was less robust in neonatal mice receiving intravenous rAAV2/1. Likewise, in juvenile mice receiving rAAV2/9, inner ear transduction was limited. These results suggest that intravenous injection of rAAV2/9 can be used in neonatal mice as an atraumatic and relatively simple method to deliver gene therapy to the cochlea.

## Results

### Intravenous delivery of rAAV2/9-CMV-eGFP leads to robust dose-dependent transgene expression in neonatal ears

To investigate the inner ear transduction profile following intravenous injection of rAAV2/9-CMV-eGFP in neonatal mice, and whether transduction efficiency could be improved in a dose-dependent manner, intravascular injections were performed via the superficial temporal vein delivering a total volume of 50 µl to neonatal mice (Fig. 1b). Two different concentrations of rAAV2/9-CMV-eGFP were administered - either 3.28 × 10^13^ (high titer) or 6.55 × 10^12^ (low titer; 1/5 of the high titer) vg/ml.

**Figure 2:**
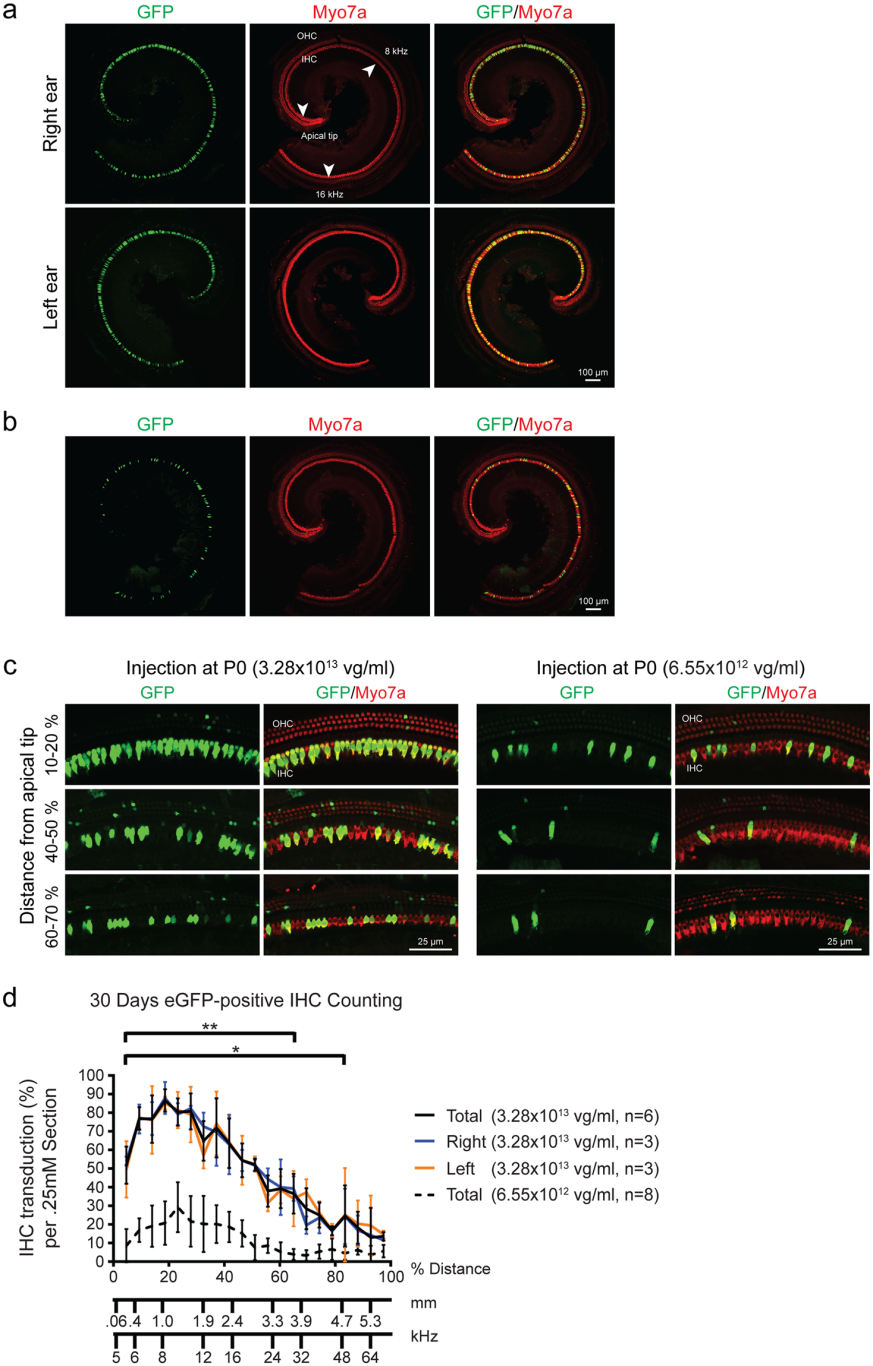
Bilateral rAAV2/9 infection via systemic inoculation is dose dependent. Injected mice (high and low dose) were sacrificed 4wks after rAAV2/9 inoculation. Ears were fixed, dissected and stained as cochlear whole mounts. All images were stained with Myo7a (red) for labeling hair cells and imaged for native eGFP (green). (**a**) 10x images of representative whole-mount apical turns from the higher-titer injected mice showing both ears. There are no differences in eGFP expression. Arrowheads show the apical tip and 8 and 16 kHz regions along the apical turn of the cochlea. (**b**) 10x images of representative whole-mount apical turns from the lower-titer injected mice. As compared to (**a**), there is a significant decrease in IHC transduction in the apical turn. (**c**) 40x magnification at the indicated position in relation to the cochlear apex. IHCs and the three rows of OHCs are shown. (**d**) The efficiency of rAAV2/9 transduction in IHCs was quantified with 20x images of whole-mount cochlea compiled into cochleograms at 4 weeks. eGFP-positive IHCs were counted in 0.25 mm segment and plotted against the distance (%) from the apex. Compared to the lower-titer injection, the higher-titer injection resulted in much stronger rAAV2/9 transduction, with similar transduction in both ears. Data are means ± SD (n, number of cochleas; *p < 0.05; **p < 0.005).

Thirty days after delivery of rAAV2/9-CMV-eGFP, whole mount sections of the membranous labyrinth were analyzed to quantitate inner hair cell (IHC) transduction (Fig. 1c and d). All injected mice demonstrated a similar transduction profile, with IHCs being the primary cell type transduced (Fig. 2a–c). All mice demonstrated strikingly similar binaural inner ear eGFP expression (Fig. 2a), with an obvious dose-dependent effect (Fig. 2b and c). The distribution of the eGFP was more robust in the apical as compared to the basal turn of the cochlea (Fig. 2c). A cochleogram plotted along the length of the cochlear duct showed that mice receiving the higher titer had significantly greater transduction of IHCs in the apical turns, with up to 96% gene transduction 1 mm distal to the apex (Fig. 2d). IHC transduction decreased in apex-to-base gradient along the length of the cochlear duct. Of note, there was no intravenous injection associated hair cell loss (Supplement Fig. 1).

### Spiral ganglion cells (SGCs) and the stria vascularis are transduced following rAAV2/9-CMV-eGFP IV injection

**Figure 3:**
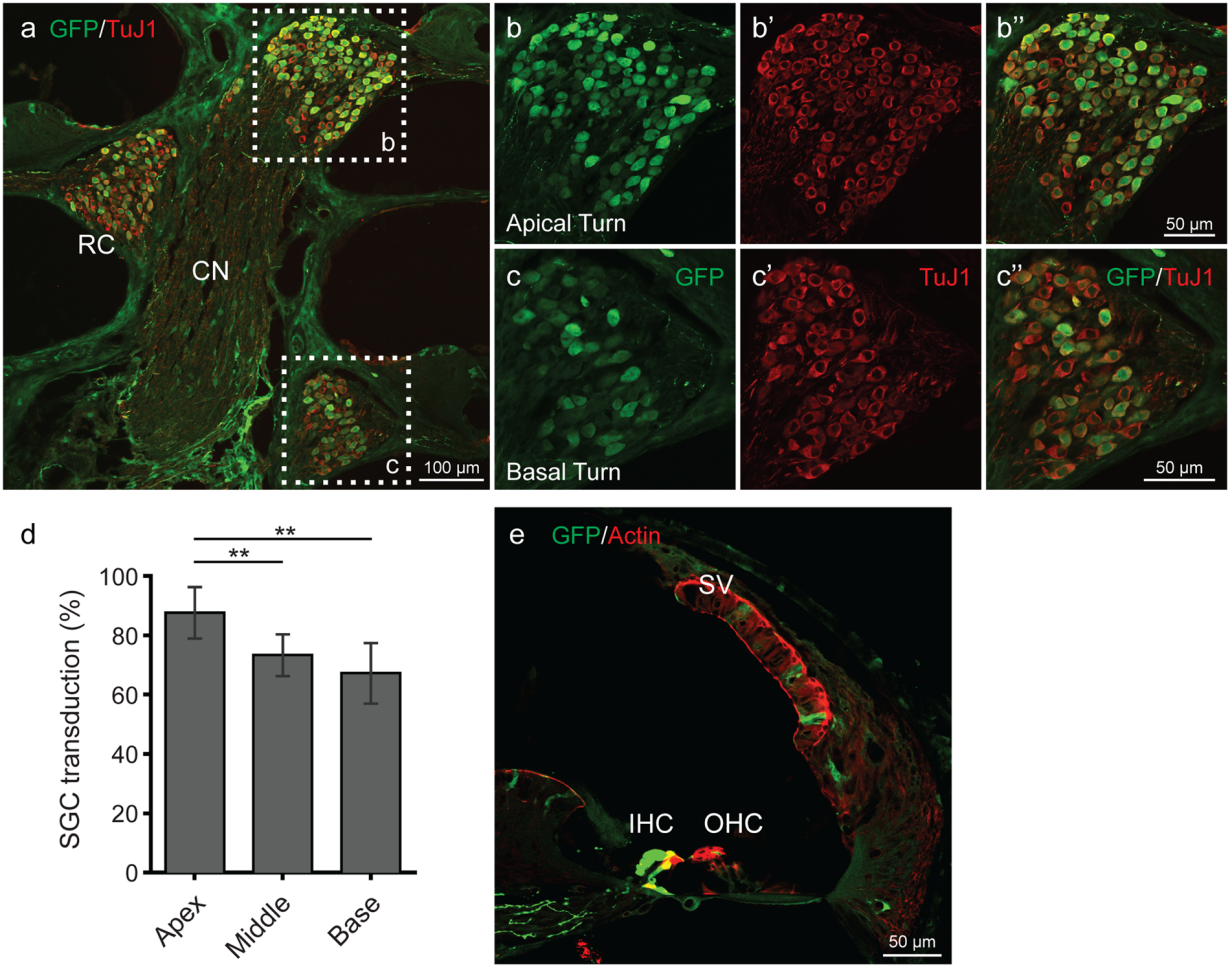
rAAV2/9 transduction in the spiral ganglion cells (SGCs) and organ of Corti 4 weeks after virus inoculation at P0–1. (**a**) Midmodiolar cross-sectional images show transduced SGCs in Rosenthal’s canal (RC) (CN, cochlear nerve). Samples were immunostained with anti-GFP (green) antibody and anti-TuJ1 (red) to label SGCs. (**b**,**c**”) Images in cross-sections of the apical (**b**–**b**”) and basal (**c**–**c**”) turns are high-magnification views of the regions marked with white dotted squares. (**d**) Percent of transduced SGCs in RC. Data are means ± SD from four sections in each of two ears. eGFP expression of SG﻿Cs﻿ in RC was greater in the apical turn than in the basal turn (*p < 0.05; **p < 0.005). (**e**) In a cross section confocal image, rAAV2/9 transduced some cells in the stria vascularis (SV). Images were immunostained with anti-GFP (green) antibody and phalloidin (red) to label filamentous actin.

To define the extent of transduction of SGCs and stria vascularis, we analyzed cochlear frozen cross sections in mice treated with the higher titer of rAAV2/9-CMV-eGFP. Robust transduction in the soma of the SGCs was observed, with more prominent expression in the apical as compared to basal turns (Fig. 3a–d). Transduction of the nerve fibers of the bipolar cells was also noted. Expression of eGFP was observed in the tissue of the stria vascularis.

### Vestibular organs are transduced following rAAV2/9-CMV-eGFP IV injection

**Figure 4:**
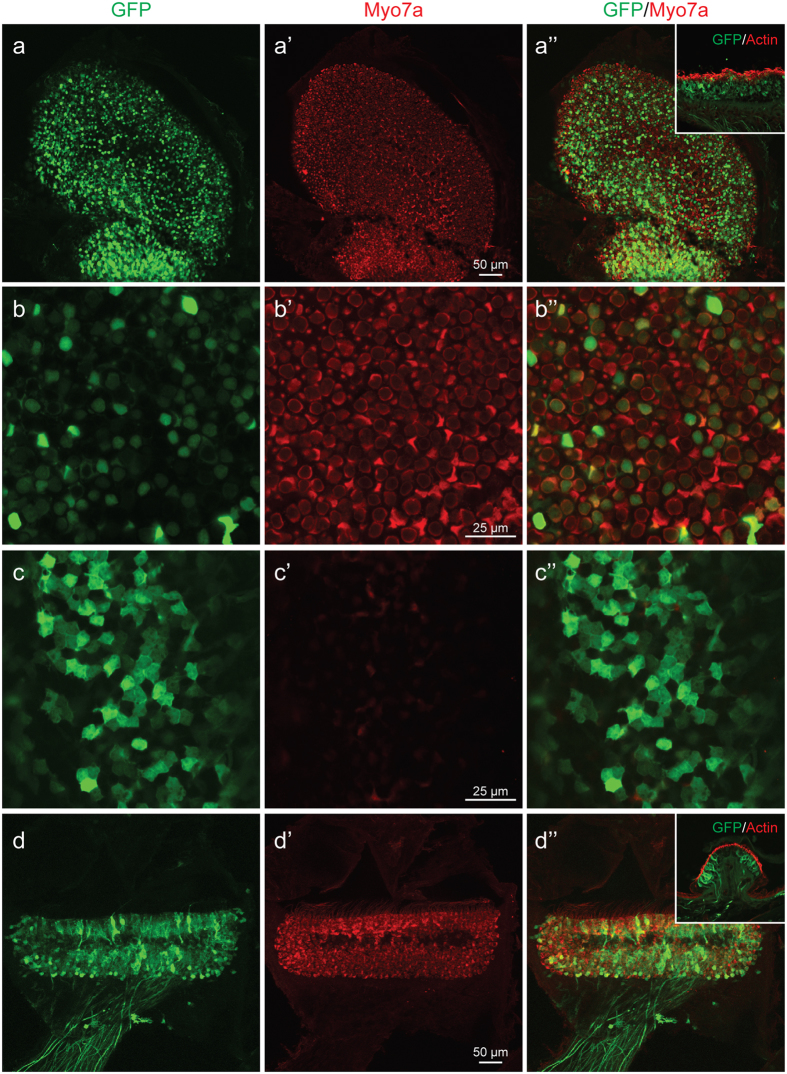
rAAV2/9 transduction in the vestibule 4 weeks after virus inoculation at P0–1. All images, except insets, were stained with Myo7a (red) for labeling hair cells (HCs) and imaged for native eGFP (green). (**a**–**a**”) Confocal images of whole mounts of the utricle. eGFP expression is evident throughout the utricle. (**b**–**b**”) High-magnification images of the HC layer show transduced HCs (eGFP-positive cells). (**c**–**c**”) High magnification images of the SC layer transduced supporting cells (eGFP-positive cells). (**d**–**d**”) Confocal images of whole mounts of the crista ampullaris (CA). eGFP expression is evident throughout the CA. Insets: Cross-sectional images of the utricle and CA. Images were stained with eGFP (green) and phalloidin (red) for labelling hair cells and filamentous actin, respectively. Note hair cell and supporting cell transduction consistent with whole mounts figures.

The vestibular organ is an equally important target for inner ear gene transfer and shares equal vasculature with the cochlea. We investigated transgene expression in the utricle and ampullaris of the anterior semicircular canal on whole mount (Fig. 1c) and frozen sections. Robust transduction in both utricles (Fig. 4a–c”) and ampullae (Fig. 4d–d”) was observed. In the utricles, the vestibular hair cells (55.2 ± 6.1%, Fig. 4b–b”) and underlying vestibular supporting cells (Fig. 4c–c”) demonstrated eGFP transgene expression. In the ampullae, eGFP expression was noted in the vestibular hair cells and vestibular nerve fibers. Treated mice did not demonstrate circling or head tilting.

### Auditory thresholds are unchanged by neonatal IV rAAV2/9-CMV-eGFP injection

**Figure 5:**
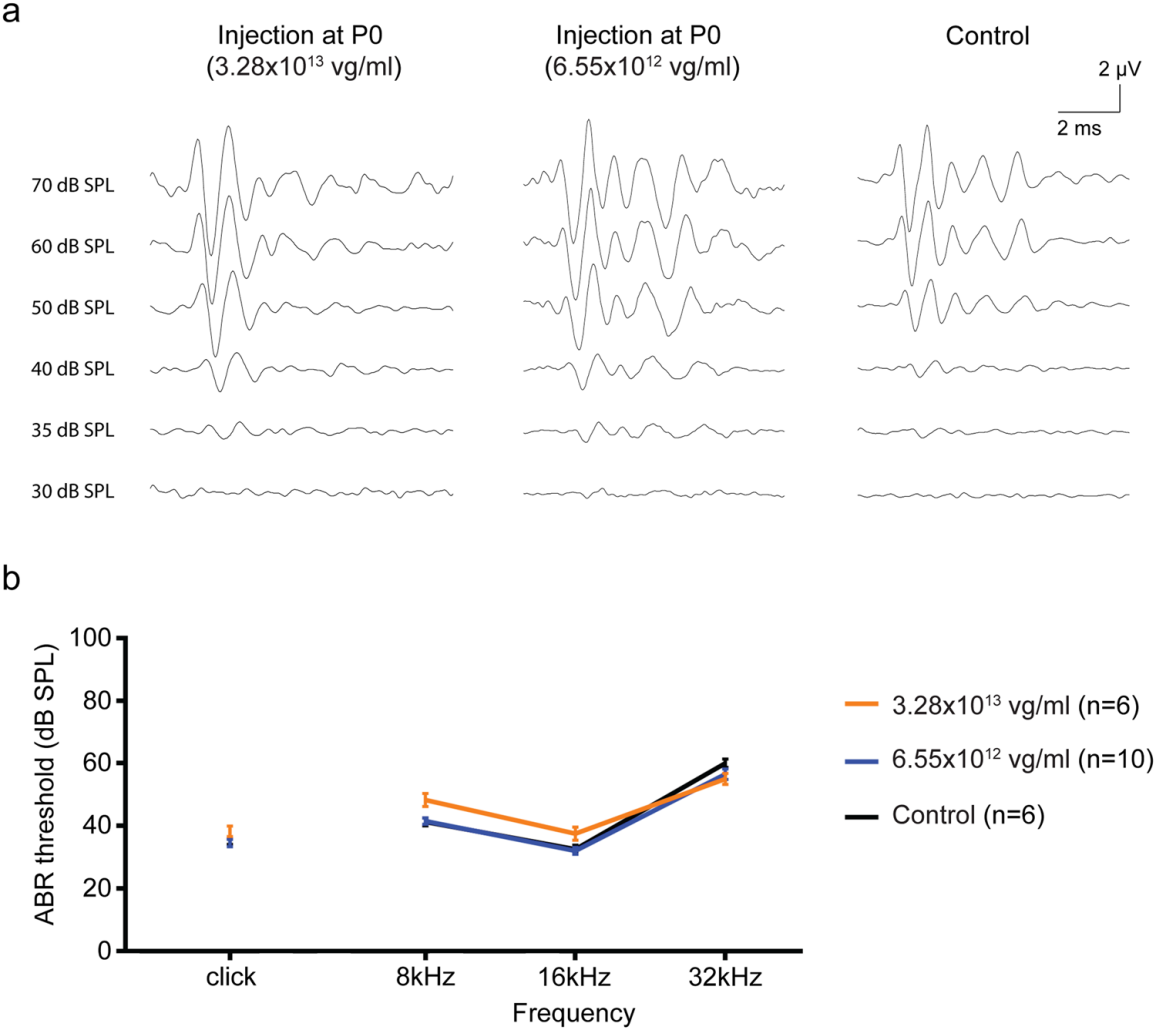
Comparison of the ABR data from P0–1 injected and control mice. (**a**) Representative ABR traces recorded from injected (high and low titer) mice 4wks after AAV2/9 inoculation at P0-1 (high and low dose) and the control 4-wk-old mice. Waveforms from injected and control mice appear similar. (**b**) Click and tone-burst ABRs show relatively normal threshold profiles in the injected mice compared with control mice at 4wks (n, numbers of ears). Slight differences of ABR threshold of less than 10 dB were observed across all frequencies. Data are means ± SEM.

We assessed auditory function by measuring auditory brainstem response (ABR) thresholds 4 weeks after intravenous injection to assess potential ototoxicity. Bilateral ears were measured in all animals from each group and in non-injected control animals. There were no statistically significant differences between ears in treated animals, and auditory performance in all treatment groups was comparable to the untreated control group (Fig. 5a and b). These results suggest that intravenous injection and IHC transduction does not alter auditory function. Likewise, treated mice did not demonstrate behavioral side effects (e.g. head tilting, weight loss, circling or ear infection) and were healthy.

### Inner ear transduction following IV injection is AAV serotype dependent

**Figure 6:**
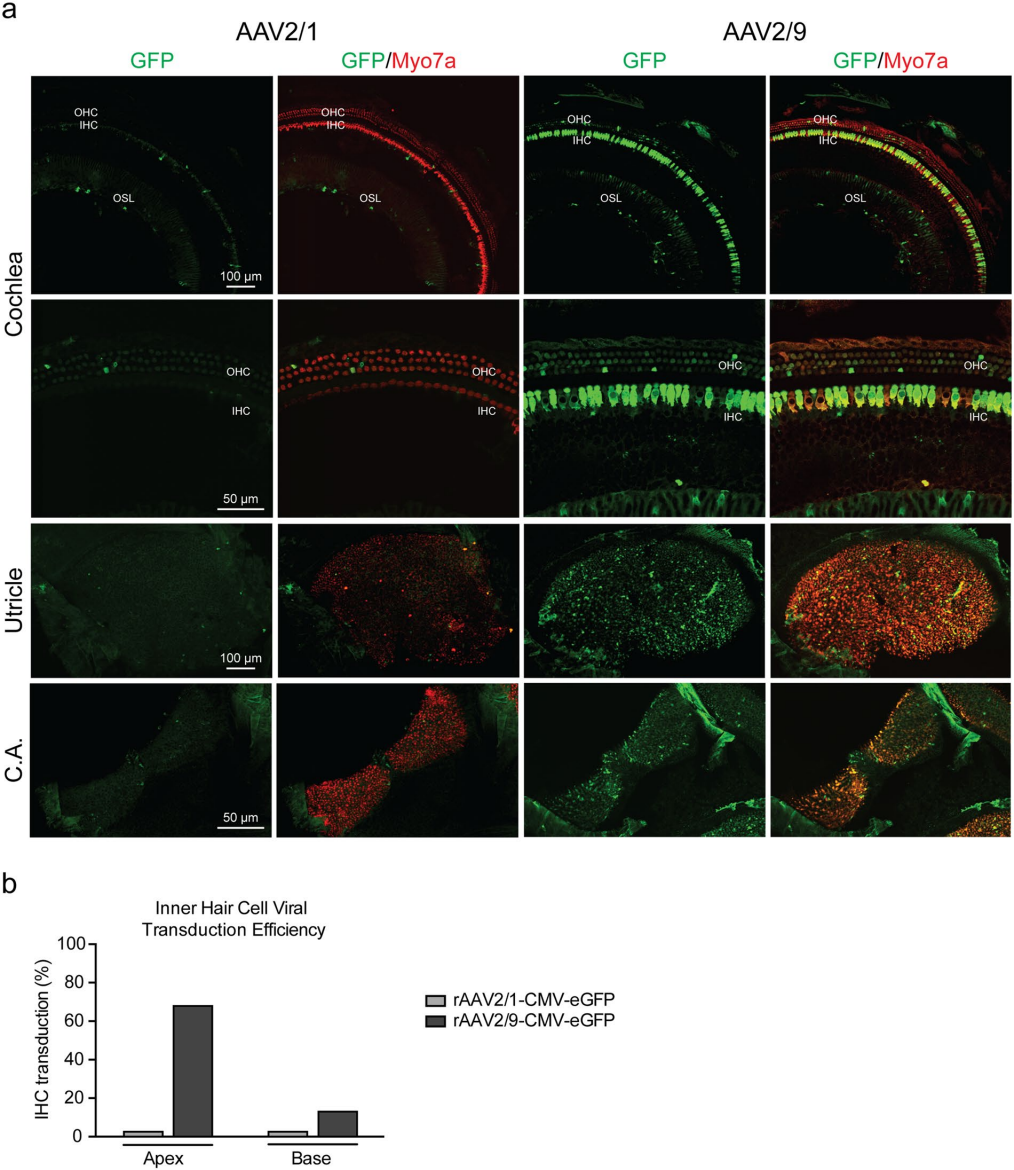
Evaluation of the Efficiency and Specificity of AAV Transduction. (**a**) Comparative transduction efficiency and specificity of rAAV2/1 and rAAV2/9 carrying a CMV-eGFP expression construct delivered to wild-type murine cochlea at P0–1 by intravenous injection. Higher resolution images in the apical turn, utricle and crista ampullaris (CA). Green represents eGFP expression and red is Myo7a for labelling of hair cells. Overlapping Myo7a and eGFP localization represents positive hair cell transduction. (**b**) The efficiency of viral transuction in IHCs was assessed in 400 µm segments in the apical and basal turns (rAAV2/1 [gray] and rAAV2/9 [black]).

We compared intravenous injection of rAAV2/1 and 2/9 using titers of 3.09 × 10^12^ vg/ml and 1.59 × 10^12^ vg/ml, respectively. With rAAV2/9-CMV-eGFP, expression of eGFP was observed in both cochlear and vestibular tissues (Fig. 6a). IHCs in apical half turns of the membranous labyrinth were primarily transduced, with sparse transduction of OHCs and supporting cells. Vestibular hair cells in the utricle and ampullaris of the anterior semicircular canal were also transduced. In comparison, when intravenous rAAV2/1-CMV-eGFP was used, expression was restricted to a few hair cells and supporting cells in both the membranous labyrinth and the vestibular organs suggesting that transduction efficiency is serotype dependent and that rAAV2/9 is superior to rAVV2/1 (Fig. 6b).

### Decreased inner ear virally-mediated gene expression following IV injection of rAAV2/9-CMV-eGFP in juvenile ages

We investigated the temporal window of efficient inner ear transduction by injecting 100 µl of rAAV2/9-CMV-eGFP at 3.28 × 10^13^ vg/ml in juvenile mice at P14–15. Because use of the superficial temporal vein is not feasible at this age, injections were completed via the external jugular vein. Ears were harvested at P30 and dissected for whole mount preparation. We found very limited transduction with few countable eGFP-positive hair cells in the apical and basal turns (Supplement Fig. 2). While the presence of eGFP-positive cells demonstrates the feasibility of inner ear transduction at this age, efficiency with rAAV2/9 is markedly reduced.

### Wide-spread transduction of the brain and skeletal muscles follows rAAV2/9-CMV-eGFP IV injection

As systemic injection of rAAV2/9 facilitates wide-spread transduction, we sought to examine the transduction profile in other organs. Gene expression was observed in the cerebral cortex and cerebellum, and in the skeletal muscle of the quadriceps, consistent with reported observations when rAAV2/9-CMV-eGFP is used for other purposes^19, 20, 28^. We found that astrocytes and Purkinje cells were transduced with higher efficiencies in neonatal mice receiving higher doses. Mice injected via the external jugular vein at P14–15 showed similar transduction profiles although efficiency was limited, similar to the changes we observed in the inner ear (Supplementary Fig. 3) and again consistent with other studies^28^. In contrast, transduction of muscle was stable in both neonatal and juvenile mice.

## Discussion

This study is the first to validate intravenous injection as a systemic approach for cochlear gene delivery. We show that if rAAV2/9 is used, widespread binaural transduction can be mediated in the cochlea IHCs, spiral ganglion neurons and vestibular hair cells. The technique is safe and does not affect auditory thresholds, although there are age- and viral serotype-dependent effects. Importantly, the procedure should be broadly applicable for cochlear gene therapy in neonatal mice with various forms of congenital deafness or vestibular dysfunction that are secondary to the pathophysiology of IHCs, spiral ganglion neurons and vestibular hair cell.

Since the first reported use of rAAVs for *in vivo* cochlear gene transfer experiments 20 years ago^29^, multiple inoculation methods have been developed to maximize efficiency and minimize iatrogenic trauma. In this study, we have shown that the intravascular injection of rAAV2/9 offers a simple and atraumatic method to deliver transgenes to the neonatal inner ear. In comparison to other delivery methods, this approach is: (1) very simple, although practice is required to successfully inject the superficial temporal vein; (2) atraumatic, as the ear is not accessed; (3) binaural, offering simultaneous transduction of both the membranous cochlea and vestibular organs; (4) reproducible; and (5) potentially applicable to all premature mice (including fetal mice via the yolk sac vein ref. 20). Disadvantages of this method include: (1) global transduction, which leads to transgene expression in off-target tissues that may result in off-target side-effects; (2) volume limitations, which should not exceed 100 µl in neonates to avoid hypervolemia; (3) transduction limitations, both in terms of cell type, cell location and animal age, which may reflect viral tropism of rAAV2/9; and (4) delivery confirmation, as successful delivery to the ear at the time of injection cannot be confirmed.

The potential feasibility of intravascular injection of AAVs for cochlear gene transfer is not intuitive. The cochlea is separated by a tight BLB, which shares many similarities with the BBB^30, 31^. It prevents molecules and viruses from entering the endolymphatic and perilymphatic spaces. Nevertheless, our data show that rAAV2/1 and 2/9 reach the cochlear duct (Fig. 6a). The presence of eGFP expression in the stria vascularis suggests that rAAV vectors may extravasate from the vasculature and enter the cochlear duct lumen by permeation of the BLB (Fig. 3e). The exact mechanism of rAAV2/9 crossing BBB into the CNS is unknown although transcytosis is thought to be associated with the crossing the endothelial barrier^24^. Further investigation is needed to determine whether these entry pathways/transport mechanisms are used in the cochlea.

We found that intravenous injection in juvenile mice provides only limited inner ear transduction, although transgene expression remains stable in tissues like muscle and cerebellum (Supplementary Figs 2 and 3). This observation is consistent with other reports demonstrating that the expression pattern of rAAV2/9 following systemic injection is influenced by age and that maturation of the BBB leads to a narrow therapeutic window for tissues like the retina^32^. Thus while the BLB reduces viral entry into membranous labyrinth in adult mice, it is possible that entry can be potentiated. For example, hyperosmolar mannitol pretreatment improves CNS transduction of AAV2 and AAVrh10 vectors by temporarily disrupting the BBB^33, 34^ although rAAV2/9 transduction is not impacted^28^. Testing various rAAV serotypes and the effect of mannitol pretreatment may widen the window for intravenous delivery of therapeutics to the inner ear.

Currently, the perilymphatic approach is used most commonly to deliver vectors into the inner ear. It is minimally invasive injection and has been clinically established for cochlear implantation. As compared to systemic injection, its main advantage is high target selectivity, which minimizes viral spread and limits transgene expression to the treated ear. Thus from a safety perspective, perilymphatic injection will likely remain the favored approach when gene-therapy-based clinical trials begin. However, relatively low transduction in the inner ear following perilymph injections has been a challenge. Recent emergence of engineered synthetic AAV vectors, including exosome-associated AAV (Exo-AAV)^35^ and ‘designer’ ancestral aav2/anc80^15, 36^ have boosted gene transduction in the inner ear, with up to 100% transduction of IHCs in neonatal. However, the risk of iatrogenic trauma during inner ear surgery remains a concern particularly in protecting intact functioning hair cells.

Our study demonstrates the feasibility of cochlear gene delivery without the necessity for direct inner ear access. In an earlier study, we showed that rAAV2/9 has a predilection for transduction of apical IHCs when injected through the round window membrane; in this study we have shown that intravenous injection provides comparable IHC transduction efficiency^27^. These similarities suggest a tropism of rAAV2/9 for apical IHCs, which is not impacted by injection method. Importantly, systemic injection makes transduction of SGCs and vestibular hair cells feasible. From a technical perspective, the systemic method is simple compared to perilymphatic and endolymphatic approaches, and can be implemented without purchasing specialized tools like micropipettes, micromanipulators and nanoinjection systems.

The global transduction associated with systemic delivery of rAAV2/9 may make this method applicable to syndromic types of hearing loss, like hereditary keratitis-ichthyosis-deafness syndrome and the Usher syndromes^37, 38^. This approach also may be useful when hearing loss is secondary to metabolic or neurodegenerative disorders, as systemic therapy could conceivably correct both the hearing loss and its underlying cause. Although unwanted off-target effects or immunological side effects raise safety concerns, the use of tissue-specific promoters such as Myo7a^39^, Pou4F3^40^ and the α9 subunit of the acetylcholine receptor^41^ may obviate these concerns.

In conclusion, this study validates intravenous injection as a systemic approach for cochlear gene delivery and shows that widespread binaural transduction is possible. The technique is easy and safe, and may be widely applicable for cochlear gene therapy in neonatal mice with various forms of congenital deafness. Although age- and viral serotype-dependent effects are seen, the advantages offered by systemic therapy justify further research to address targeted transduction and efficiency in adult cochlear tissue.

## Materials and Methods

### Ethics Approvals

All experiments were approved by the University of Iowa Institutional Biosafety Committee (IBC; rDNA Committee; RDNA Approval Notice #100024) and the University of Iowa Institutional Animal Care and Use Committee (IACUC; Protocol #06061787) and performed in accordance with the NIH Guide for the Care and Use of Laboratory Animals.

### Virus Production

AAV viral vectors were prepared by the Gene Vector Core facility at the University of Iowa using the triple transfection method or baculovirus system as described^42^. Single-stranded recombinant AAV serotypes (rAAV2/1 and rAAV2/9) contained a transgene cassette of CMV-driven eGFP. For the dose-dependency study 50 µl of rAAV2/9 viral vector at 3.28 × 10^13^ (high titer) or 6.55 × 10^12^ (low titer; 1/5 of the high titer) were administered. Viral titers used in intravascular injections comparison study were diluted in sterile saline to concentrations of 3.09 × 10^12^ vg/ml for rAAV2/1 and 1.59 × 10^12^ vg/ml for rAAV2/9. Virus aliquots were stored at −80 °C and thawed before use.

### Animal Model and Viral Inoculation

Murine experiments were conducted using wild-type inbred C3HeB/FeJ mice purchased from the Jackson Laboratory. Neonatal mice were operated on at P0–1. Mice were placed in a container with crushed ice for 3 to 5 minutes until the onset of hypothermal anaesthesia could take effect. Intravascular injections were performed via the superior temporal vein (Fig. 1a). A total of 50 µl of each viral vector at 3.28 × 10^13^ (high titer) or 6.55 × 10^12^ (low titer; 1/5 of the high titer) vg/ml with 2.5% fast green dye (Sigma-Aldrich, St. Louis, MO) was loaded into a 30-gauge syringe. Following canalization of the vein, the viral vector was slowly injected (25 µl/sec); upon successful injection mice turned green almost immediately. After the injection, neonatal mice were placed on a heating pad for recovery and rubbed with bedding before being returned to their mother^43^.

Mice at P14 were anesthetized with intraperitoneal ketamine and xylazine at 100 mg and 10 mg per kg of body weight, respectively. They were placed in a recumbent position on a heating pad, and a small incision was made lateral to the ventral midline from the pectoral muscle to the lower neck. After the incision, an external jugular vein was exposed. A total of 200 µl of each viral vector at 3.28 × 10^12^ vg/ml was delivered into the jugular vein using a 30-gauge needle.

### Auditory Testing

All mice were anesthetized as described above. Sound stimuli were generated using an RZ6 auditory processor driving two MF1 Multi-Field Magnetic Speakers (Tucker-Davis Technologies, Alachua, FL). Closed field transmission of the sound waves generated by the MF1 speakers was achieved by connecting 2-inch lengths of plastic tubing from the speakers to an ER-10B + probe microphone (Etymotic Research, Elk Grove Village, IL). A speculum mounted on the ER-10B + probe microphone was inserted into the auditory canal of the tested ear. The speculum formed a tight seal against the ear canal completing the closed field transmission of auditory stimuli.

The setup was calibrated using a 0.028 cm^3^ cavity to approximate the size of the mouse ear canal. All recordings were conducted from both ears of all animals on a 37-degree heating pad. Clicks were square pulses 100ms in duration and tone bursts were 3ms in length at distinct 8, 16, and 32 kHz frequencies. ABRs were measured with BioSigRZ (Tucker-Davis Technologies) for both clicks and tone bursts, adjusting the stimulus levels in 5-decibel (dB) increments between 10–90 dB sound pressure level (SPL) in both ears. Electrical signals were averaged over 512 repetitions. ABR threshold was defined as the lowest sound level at which a reproducible waveform could be observed. Responses from wild-type inbred C3HeB/FeJ mice at 4 weeks were used as controls. ABRs were measured 4 weeks after the intravenous injection.

### Fluorescence Microscopy and Immunohistochemistry

Bilateral inner ears, brain, cerebellum and skeletal muscle were harvested 4 weeks after the intravenous injection. Deeply anesthetized animals were perfused transcardially with 4% paraformaldehyde for 15 min. Each tissue was locally perfused and fixed in 4% paraformaldehyde for 1hr, rinsed in PBS, and stored at 4 °C in preparation for immunohistochemistry. Inner ears used in whole mount preparations for the comparison study (Fig. 6) were stained for eGFP to enhance signal, otherwise native eGFP signal were observed. Following infiltration using 0.3% Triton X-100 for 30 min and blocking with 5% normal goat serum for 1hr, tissues were incubated with rabbit polyclonal Myosin-VIIA antibody (Proteus Biosciences Inc., Ramona, CA) diluted 1:200 in PBS for 1hr. Fluorescence-labeled goat anti-rabbit IgG Alexa Fluor 568 in 1:500 dilution was used as a secondary antibody (Thermo Fisher Scientific, Rockford, IL) for 30 min. For cryo-sectioning, cochleae were decalcified in 120 mM EDTA for 2 days, cryoprotected in 15% and 30% sucrose, and embedded in OCT solution. 14μm midmodiolar cryosections were prepared and immunohistochemistry was performed. Following infiltration using 0.3% Triton X-100 for 30 min and blocking with 5% normal goat serum for 1hr, we incubated the tissues in following primary antibodies diluted in PBS: 1) mouse monoclonal antibody to eGFP (Millipore, Temecula, CA) at 1:200; 2) rabbit polyclonal antibody to TuJ1 (BioLegend, San Diego, CA) at 1:1000 overnight. We used fluorescence-labeled goat anti-mouse IgG Alexa Fluor 488 and 647 secondary antibodies at 1:500 dilution. Filamentous actin was labeled by a 30 min incubation of phalloidin conjugated to Alexa Fluor 568 (Thermo Fisher Scientific) in 1:100 dilution (exceptions: cortex, cerebellum, skeletal muscle). Specimens were mounted in ProLong® Diamond Antifade Mounting Media (Thermo Fisher Scientific) and observed with a Leica TCS SP8 confocal microscope (Leica Microsystems Inc., Bannockburn, IL).

### Cell counts and transduction efficiency analysis

Images of whole mount and frozen sections, as described above, were used to count transduced hair cells and SGCs using the ImageJ program (NIH Image). Images were prepared using Adobe Photoshop CC to meet equal conditions.

Transduction efficiency was evaluated by counting eGFP-positive cells from the following three cell types: (1) IHCs - to evaluate IHC transduction we counted eGFP-positive cells across a 250 µm radius from the apical turn to the basal turn. Hair cells with overlapping Myo7a and eGFP signals were considered transduced; (2) Utricular HCs - HC transduction in the utricle was assessed by taking images of three non-overlapping views (100 µm × 100 µm per view) randomly captured from the HC layers of each utricle (n = 6). We then counted eGFP-positive hair cells. Hair cells with overlapping Myo7a and eGFP signals were considered transduced; 3) SGCs - transduction of SGCs of the Rosenthal’s canal were counted by capturing views from the apex, middle and base of Rosenthal’s canal for each section. A total of 8 sections were evaluated. Hair cells with overlapping TuJ1 and eGFP signals were considered transduced. For each group, the total number of cells (IHCs, utricular HCs or SGCs) was also counted; transduction was then expressed as a percentage of the total.

Hair cell survival was assessed by counting the number of surviving hair cells at the p30 time point. IHCs and OHCs with positive Myo7a were counted; any segments that contained dissection-related damage were omitted from the analysis. IHC and OHC survival was quantified with images of whole-mount cochleae compiled into cochleograms.

### Statistical Analysis

Statistical analysis of ABR and cell-counting data was completed in R with two-sample t tests for samples of equal variance a P value < 0.05 was considered significant. Samples with unequal variance were compared with Welch two-sample t tests. Sample variance was determined with F tests comparing two variances.

## Electronic supplementary material

Supplementary information
