# Supplementary material for: Intravenous rAAV2/9 injection for murine cochlear gene delivery
Source: Sci Rep. 2017 Aug 29;7:9609. doi: 10.1038/s41598-017-09805-x (PMC5575199; doi:10.1038/s41598-017-09805-x)
Supplement: Supplementary file 1 — Supplementary information [file 41598_2017_9805_MOESM1_ESM.pdf]

Supplementary Information

Intravenous rAAV2/9 injection for murine cochlear gene delivery

Seiji B. Shibata[1,2†], Hidekane Yoshimura [2,3†], Paul T. Ranum[2,4], Alexander T. Goodwin[2] and Richard J.H. Smith[1,2,4,5*]

[1] Department of Otolaryngology - Head and Neck Surgery, Carver College of Medicine, University of Iowa, Iowa City, IA 52242, USA

[2] Molecular Otolaryngology and Renal Research Laboratories, Carver College of Medicine, University of Iowa, Iowa City, IA 52242, USA

[3] Department of Otorhinolaryngology, Shinshu University School of Medicine, Matsumoto, Nagano 390-8621, Japan

[4] Interdisciplinary Graduate Program in Molecular & Cellular Biology, The University of Iowa Graduate College, University of Iowa, Iowa City, IA 52242, USA

[5] Iowa Institute of Human Genetics, Carver College of Medicine, University of Iowa, Iowa City, IA 52242, USA

[†] These authors contributed equally

Supplementary Figure Legends

Supplemental Figure 1: Percentage of surviving IHCs and OHCs (relative to WT) across a 250µm radius from the apical turn to the basal turn.

Supplemental Figure 2: Efficiency and specificity of rAAV2/9 transduction at P14 by intravenous injection (a,b) rAAV2/9 showed relatively weak eGFP expression in the apical (a) and basal (b) turn. (c) shows high resolution image of the areas in the boxed portions of a and b. Overlapping Myo7a (red) and eGFP (green) localization represents positive hair cell transduction.

Supplemental Figure 3: Transduction pattern in brain and skeletal muscle following systemic injection of rAAV2/9. (a-c) cortex; (d-f) cerebellum; (g-i) skeletal muscle (quadriceps). Sections from injected mice at P0-1 (high and low dose) and P14 were immunofluorescently labelled with anti-GFP antibody. (a-f) In the confocal images of brain sections, mice had eGFP expression in cortical astrocytes (arrows) and Purkinje cells (arrowheads). The expression level of eGFP positive cells was higher in the injected mice at P0-1 (high dose) compare to at P0-1 (low dose) and P14. (g-i) The transduction pattern in the quadriceps was not affected by age at injection.

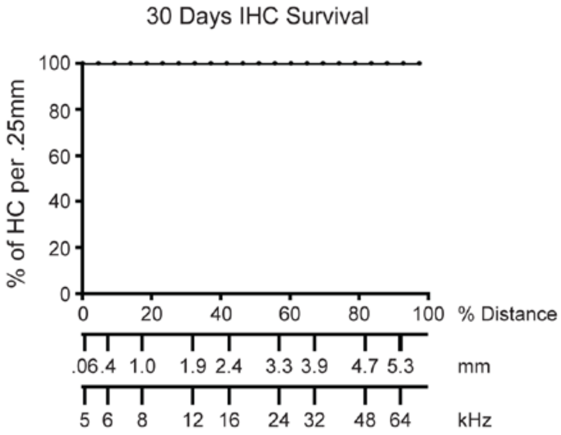
30 Days IHC Survival
% of HC per .25mm
100
80
60
40
20
0
0
20
40
60
80
100
% Distance
.06
.4
1.0
1.9
2.4
3.3
3.9
4.7
5.3
mm
5
6
8
12
16
24
32
48
64
kHz

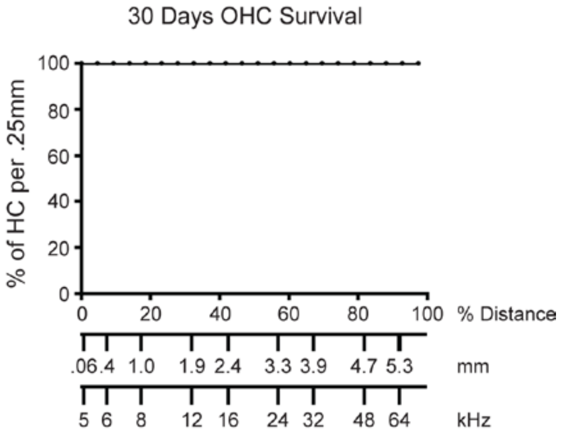
30 Days OHC Survival
% of HC per .25mm
100
80
60
40
20
0
0
20
40
60
80
100
% Distance
.06
.4
1.0
1.9
2.4
3.3
3.9
4.7
5.3
mm
5
6
8
12
16
24
32
48
64
kHz

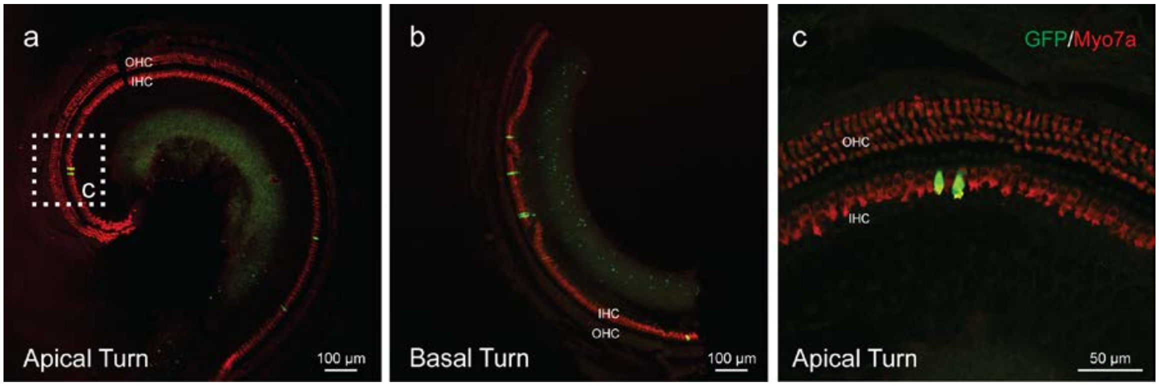
a
OHC
IHC
c
Apical Turn
100 µm
b
IHC
OHC
Basal Turn
100 µm
c
GFP/Myo7a
OHC
IHC
Apical Turn
50 µm

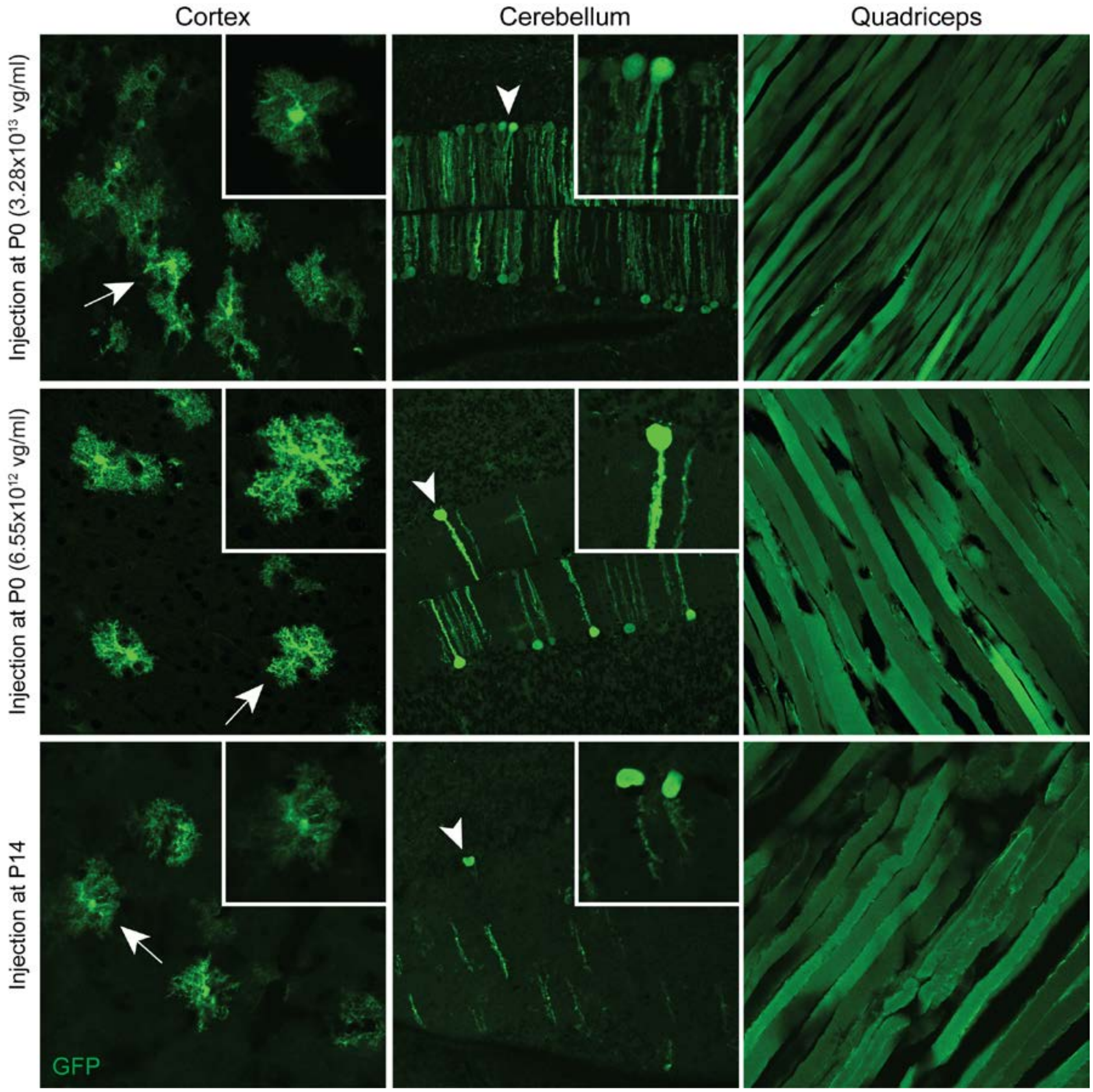
Cortex
Cerebellum
Quadriceps
Injection at P0 (3.28x10^13 vg/ml)
Injection at P0 (6.55x10^12 vg/ml)
Injection at P14
GFP
